# Differentially expressed serum proteins from obese Wistar rats as a risk factor for obesity-induced diseases

**DOI:** 10.1038/s41598-020-69198-2

**Published:** 2020-07-24

**Authors:** Kwazikwakhe Bethuel Gabuza, Nicole Remaliah Samantha Sibuyi, Mmabatho Peggy Mobo, Abram Madimabe Madiehe

**Affiliations:** Department of Biotechnology, University of the Western Cape, Bellville, Cape Town, 7535 South Africa

**Keywords:** Biochemistry, Biotechnology, Computational biology and bioinformatics, Biomarkers

## Abstract

Obesity is a chronic disease that negatively affects life expectancy through its association with life-threatening diseases such as cancer and cardiovascular diseases. Expression proteomics combined with in silico interaction studies are used to uncover potential biomarkers and the pathways that promote obesity-related complications. These biomarkers can either aid in the development of personalized therapies or identify individuals at risk of developing obesity-related diseases. To determine the serum protein changes, Wistar rats were fed standard chow (low fat, LF), or chow formulated high fat (HF) diets (HF1, HF2 and HF3) for 8 and 42 weeks to induce obesity. Serum samples were collected from lean and obese rats at these time points. The serum samples were precipitated using trichloroacetic acid (TCA)/acetone and analyzed by 2-Dimensional SDS-PAGE. Serum protein profiles were examined using mass spectrometry (MS)-based proteomics and validated by western blotting. Protein–protein interactions among the selected proteins were studied in silico using bioinformatics tools. Several proteins showed differences in expression among the three HF diets when compared to the LF diet, and only proteins with ≥ twofold expression levels were considered differentially expressed. Apolipoprotein-AIV (APOA4), C-reactive protein (CRP), and alpha 2-HS glycoprotein (AHSG) showed differential expression at both 8 and 42 weeks, whereas alpha 1 macroglobulin (AMBP) was differentially expressed only at 8 weeks. Network analysis revealed some interactions among the proteins, an indication that these proteins might interactively play a crucial role in development of obesity-induced diseases. These data show the variation in the expression of serum proteins during acute and chronic exposure to high fat diet. Based on the expression and the in-silico interaction these proteins warrant further investigation for their role in obesity development.

## Introduction

Obesity, defined by a body mass index of ≥ 30 kg/m^2^, is a global epidemic affecting both developed and developing countries^1^. Despite the major progress made in regulating energy balance and understanding the molecular mechanisms leading to the development of obesity in both human and animal studies, no safe or effective treatment has yet been found^1^. Obesity is a chronic disease that is clinically managed by lifestyle modification, pharmacotherapy, and surgery. Failure to maintain a healthy body weight has been associated with development of chronic diseases such as cardiovascular diseases, type 2 diabetes (T2D) and some forms of cancer^2–4^. These diseases pose a major health threat as they are considered the top ten leading causes of death worldwide^5^. Therefore, identification of alternative approaches that can unravel the biochemical and physiological processes that occur during development of obesity and progression to chronic diseases are urgently needed. These might give insights on obesity and obesity-induced diseases, uncover ways to prevent their development in susceptible individuals, and improve on the current treatment strategies.

Serum plays a major role in diagnosis and monitoring of many diseases through immunological assays. Serum contains many proteins originating from various tissues and cells within the body^6^, and serve as an attractive source for biomarker discovery as it can reflect the molecular changes taking place during the development of obesity and progression to chronic diseases of lifestyle^7^. These proteins can be used as potential biomarkers or targets for the targeted treatment of obesity, diagnosis and/or identification of individuals at risk of developing obesity-induced diseases. Obesity is a multifaceted disease, as a result a single biomarker cannot entirely describe all the pathways affected. Several adipokines, cytokines, metabolites, and microRNAs are implicated in obesity-induced diseases, some are dysregulated in more than one disease while some show potential as disease-specific biomarkers. Their expression levels can identify individuals with or at risk of developing obesity-related diseases such as inflammatory diseases^8^, diabetes^9^, CVDs^10^ and metabolically unhealthy obese people^11^.

Proteomics has been especially helpful in biomarker discovery for chronic diseases, not limited to cancer^12^, and obesity^11,13^. Proteomics provides a platform to study the entire proteome in a given sample, and allows for qualitative and quantitative protein profile that can discriminate between healthy and diseased samples^14^. This study aimed to use proteomics combined with bioinformatics to identify the serum proteome response to HF diet induced obesity in Wistar rats and use in silico analysis to determine potential interactions of the identified proteins.

## Results

### Dietary fatty acid composition

Wistar rats were subjected to LF as a normal diet and three different HF diets to induce obesity, distribution of fatty acids (FAs) among the four diets was analyzed by GC MS. The LF diet had significantly higher amounts of unsaturated long-chain FA content than the three HF diets as illustrated in Table 1. The LF diet contained 75% unsaturated and 25% saturated FAs. In contrast, the HF1-3 diets had higher percentage of saturated FAs (56%, 50% and 49%, respectively). The HF3 diet had the highest content of saturated FAs, followed by HF1 and HF2. The palmitic acid and stearic acid were the highest saturated FAs in all the HF diets. The HF1 diet contained two folds of short chain saturated FAs (8% myristic acid) than the other two HF diets and the LF diet. The content of oleic acid was not significantly different across the four diets.

**Table 1:** Analysis of FA content in the four diets by GC MS.

Names of fatty acids (FAs)	LF diet (%)	HF diet 1 (%)	HF diet 2 (%)	HF diet 3 (%)	Type of FAs
α-linoleic acid (18:2)	1	0	0	1	Unsaturated
γ-linoleic acid (18:3)	4	1	1	1	Unsaturated
Arachidic acid (20:0)	1	1	1	1	Saturated
Palmitoleic acid (16:1)	1	1	2	2	Unsaturated
Mysritic acid (14:0)	4	8	3	3	Saturated
Palmitic acid (16:0)	17	32	24	23	Saturated
Stearic acid (18:0)	7	15	22	22	Saturated
Oleic acid (18:1)	27	33	39	37	Unsaturated
Linoleic acid (18:2)	42	9	9	11	Unsaturated

*FA* fatty acid, *GC MS* least squares, *LF* low fat, *HF* high fat.

### Effects of HF diets on body weight and food intake

**Figure 1:**
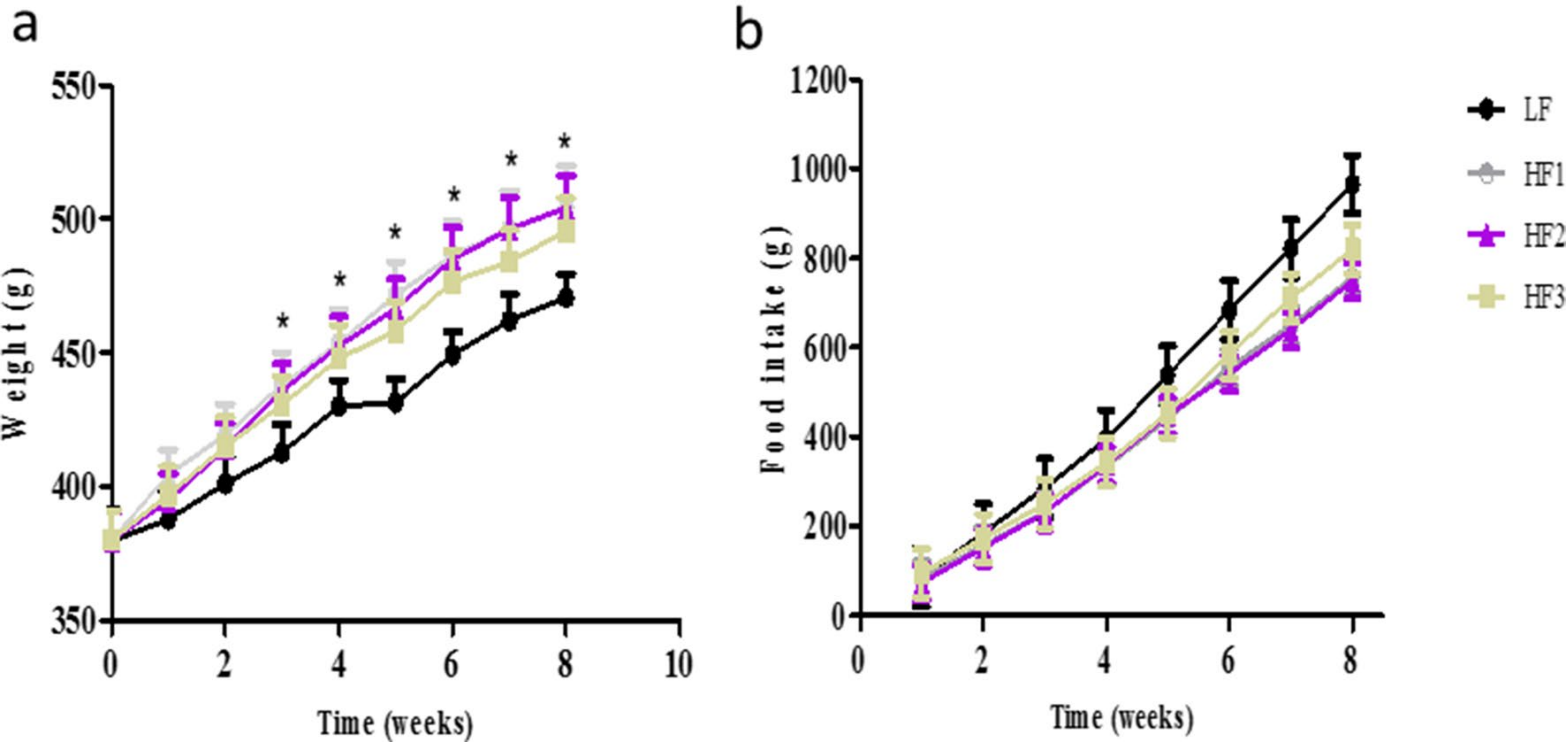
Rat body weights (**a**) and cumulative food intake (**b**) was measured weekly for 8 weeks during induction of obesity. *Represents *p* < 0.05.

A rat model of diet-induced obesity (DIO) was initially established by comparing the effects of three HF diets on body weight gain, development of obesity and serum proteome expression. As depicted in Fig. 1a, the three HF diet groups gained more weight than the LF group throughout the period of the study (8 weeks). The differences in the body weights were noticeable as early as the first week on the HF diets when compared to the rats on the LF diet. The HF1 and HF2 diets caused more bodyweight gain within 8 weeks than the HF3 diet. The difference between the LF and HF groups was statistically significant. A similar trend was observed with the chronic HF feeding (42 weeks) as that in 8 weeks, where rats on the HF1 diet gained significantly more body weight compared to rats on the LF diet after 42 weeks, 302.7 ± 67.9 g vs. 236.1 ± 11.9 g, respectively (data not shown).

To determine whether obesity (body weight gain) was caused by hyperphagia (increase in food intake), the cumulative food intake was assessed. As shown in Fig. 1b, the LF group consumed more food per gram than the three HF groups; and this was evident from the third week on their respective diets. Due to caloric density of the HF and LF diets (1 g of diet was equivalent to 5.54, 4.01, 3.85 and 2.67 cal for HF1, HF2, HF3 and LF diets, respectively), the HF 1 group had higher energy intake followed by HF2, HF3 and LF groups. These calories were calculated based on the nutritional information of the diet provided by the supplier using the 4–4-9 formula for calories from carbohydrates, protein and fat, respectively.

### Fat distribution and adiposity index

**Figure 2:**
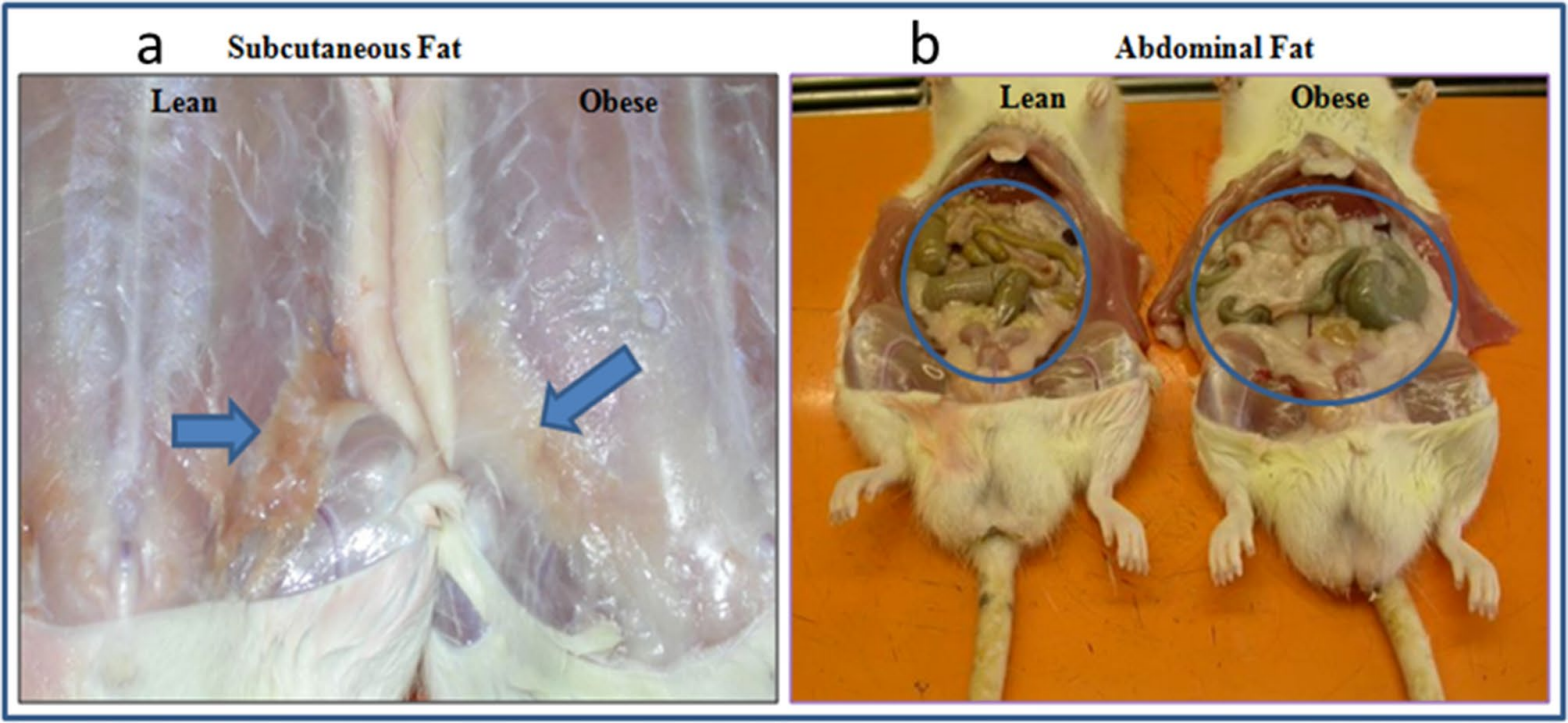
Fat distribution in the subcutaneous (**a**) and omental (**b**) fat depots of the lean (LF) and obese (HF1) rats.

The fat pads (inguinal, perirenal, retroperitoneal) contributed a significant amount to the total body weight, as shown in Fig. 2. The fat mass was especially higher in the subcutaneous and abdominal fat depots of the HF1 (obese) group when compared to the LF (lean) group. No significant difference was visually observed between the fat depots of rats on the HF2, HF3 and the LF diets after 8 weeks (data not shown).

**Figure 3:**
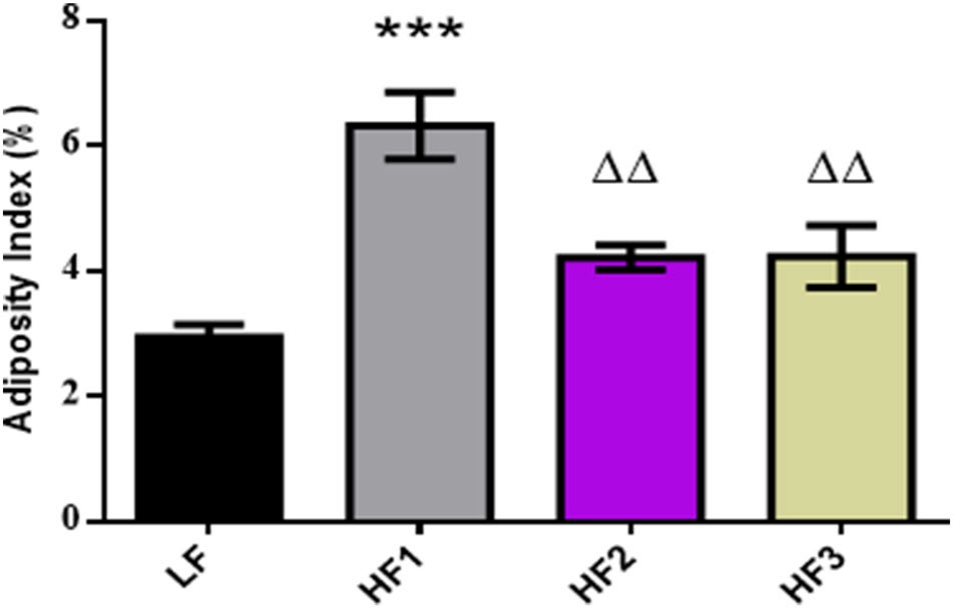
Adiposity index among HF and LF groups at 8 weeks. The AI difference was statistically significant for the HF 1 group compared to the LF, HF 2 and HF 3 groups. ***p < 0.0001 vs. LF, ∆∆*p* < 0.01 vs. HF 1.

The adiposity index (calculated as the percentage of the sum of all the white adipose tissue mass in relation to the final rat body weight, was significantly higher in the HF1 group compared to the HF2, HF3 and LF groups as shown in Fig. 3. The adipose tissue weights for the HF1 group contributed 6.3% whereas HF2 and HF3 contributed 4% and LF contributed 3%, respectively, to the final body weight. The difference between LF, HF2 and HF3 were not statistically significant.

### Analysis of serum proteome using MS-based proteomics

**Figure 4:**
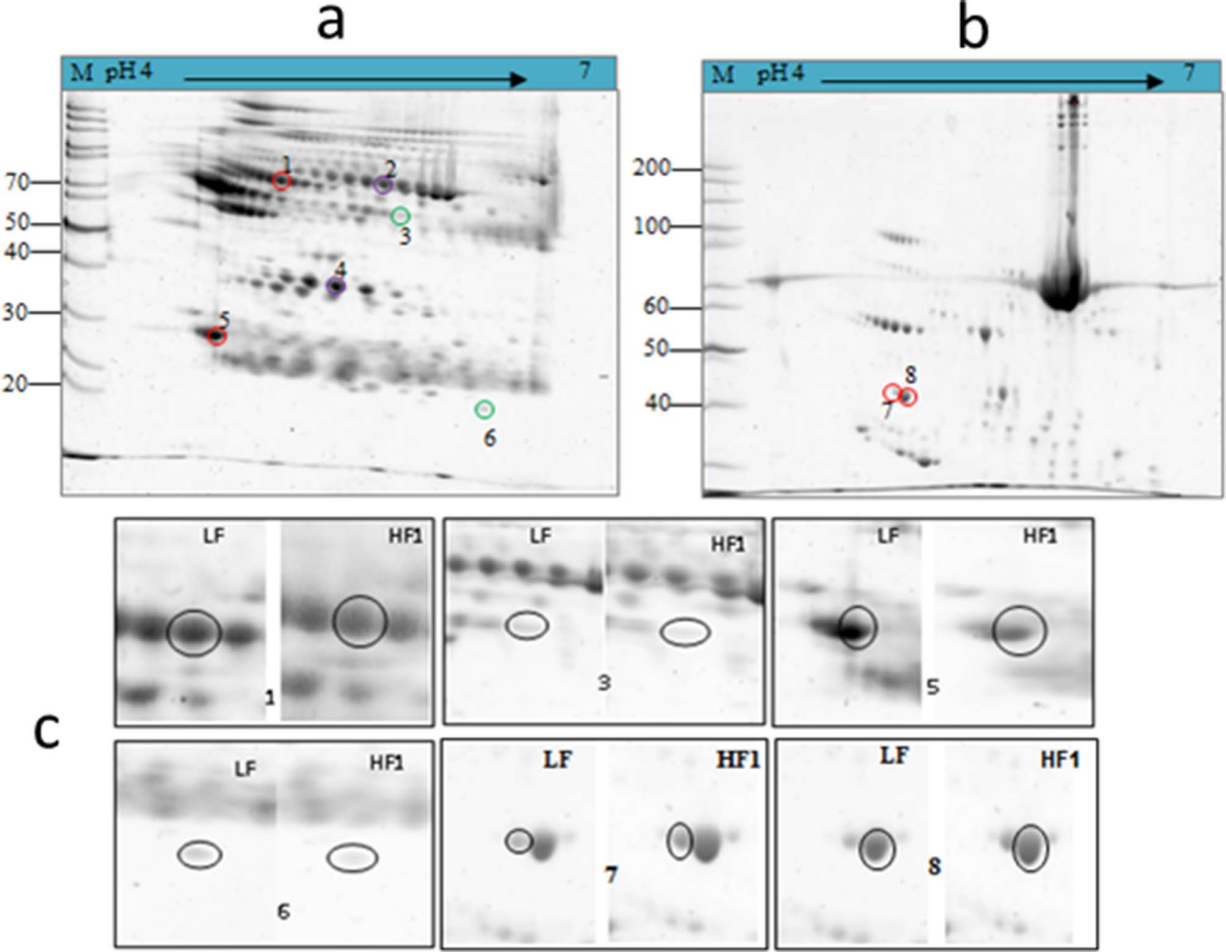
Representative 2D gel images from obese rat serum compared to lean rats. 2D gel maps of albumin-depleted (**a**) and albumin-containing (**b**) serum samples. Magnified images of differentially expressed protein spots between LF and HF samples (**c**). The circles indicate spots that were found to be significantly dysregulated in the HF group when compared to the LF group.

In this study, MS-based proteomics was used to determine the changes in protein profiles in sera obtained from lean and DIO rats. Serum albumin was removed in order to allow the detection of potential biomarkers which could be present in low abundance. Depletion of albumin from serum samples by TCA/acetone precipitation resulted in resolution of ~ 80 spots in both the albumin-depleted and albumin-containing samples as shown in Fig. 4a,b. Several spots showed significant variations between the control and HF groups. Figure 4a,b show representative 2D gel images from albumin depleted (supernatant) and albumin-containing (pellet) serum samples of obese rats, respectively. The proteins spots that are significantly dysregulated are highlighted by coloured circles, red circles indicate spots with altered expression at both 8 and 42 weeks, while the purple circles show altered expression at 8 weeks, and the green circles at 42 weeks.

Spot nos. 2, 3, 7 and 8 were found to be up-regulated and Spot nos. 1, 4, 5 and 6 were down-regulated in the HF group. Ten spots showed differential expression that was statistically significant in the albumin-containing samples (Fig. 4b), and only two of the spots showed expression levels that was ≥ twofold. The identities of the selected spots are listed in Table 2. The protein spots 1–4 in the albumin-depleted sample (Fig. 4c) were identified by MS as alpha 2-HS glycoprotein (AHSG, Fetuin-A), HPX, CRP, and AMBP, respectively. The two spots (Spot no. 7 and 8) in the albumin-containing sample were identified as APOA4 (Fig. 4c). The expression of CRP was upregulated, and AHSG was reduced in the HF groups at both 8 and 42 weeks; whilst AMBP showed reduced expression only at 8 weeks and no significant difference at 42 weeks. The expressions of HPX and APOA4 were significantly upregulated at 8 weeks, and the upregulation of APOA4 persisted up to 42 weeks.

**Table 2:** Protein spots that were differentially expressed in the serum of obese rats.

Spot no.	Protein name	NCBI accession No	Nominal mass (kDa)	Calculated pI	Sample	Time (weeks)	Expression in HF
1	Alpha 2-HS glycoprotein	Q7TP75	68	4.2	Supernatant	8/42	Upregulated
2	Hemopexin	Q5BKB4	51.3	7.58	Supernatant	8	Upregulated
3	C-reactive protein	A42579	25.5	4.89	Supernatant	8/42	Upregulated
4	Alpha 1 macroglobulin	Q63041	16.7	6.46	Supernatant	8	Downregulated
7	Apolipoprotein AIV	Q5BK92	44.5	5.18	Pellet	8/42	Upregulated
8							

### Validation of protein expression

**Figure 5:**
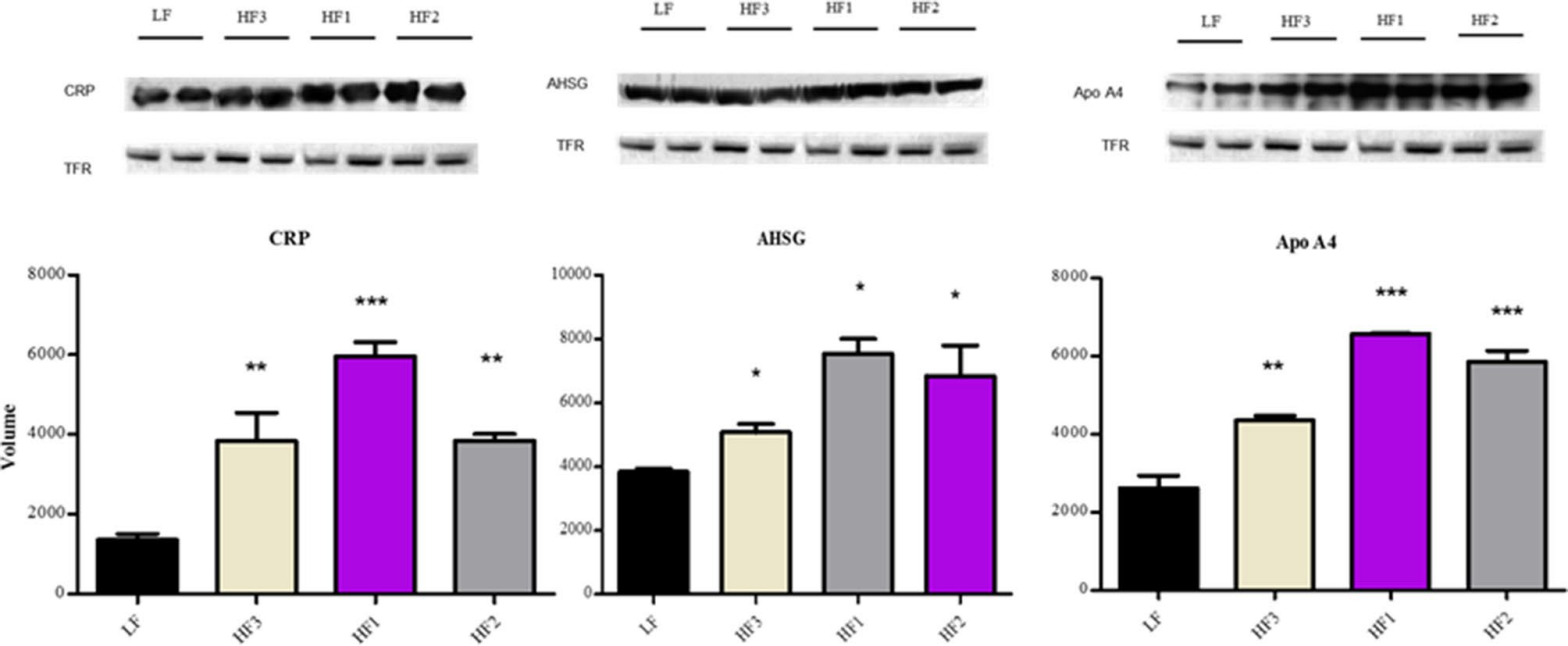
Validation of protein expression on the serum samples from lean and obese rats at 8 weeks. CRP, AHSG and ApoA4 expression was compared among the four groups by Western blot analysis and quantified by Image J software. TFR was used as a loading control. *p < 0.05 vs. LF, ***p < 0.0001, **p < 0.001.The images were cropped from the original blots (see Supplementary Data).

Expression levels of CRP, AHSG and APOA4 were validated by western blot analysis at baseline (0) and termination (8 and 42 weeks). TFR was used as an internal loading control^15^. As shown in Fig. 5, the expression levels between LF and HF groups were not statistically different at baseline (0 weeks), significant changes were observed after HF diet feeding. However, the expression levels of CRP, AHSG, and Apo-IV were upregulated in the three HF groups at 8 weeks.

### Protein and gene interactions

**Figure 6:**
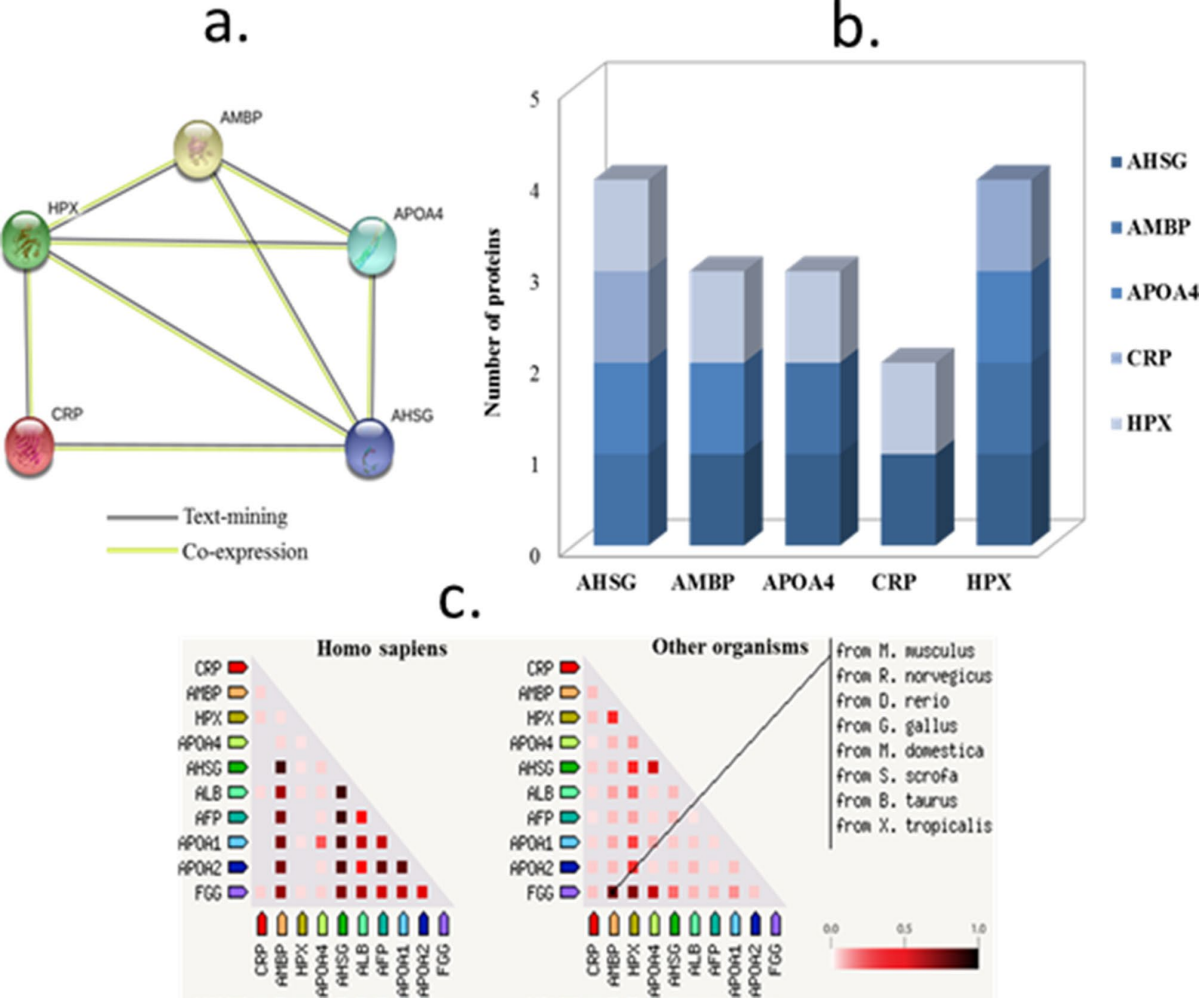
Protein–protein interactions among the differentially expressed proteins. The networks were generated in String database show to co-expression between proteins (**a**, **b**). Association between the genes are based on co-expression of homologs in *Homo sapiens* and other species (**c**). Co-expression scores are based on RNA expression patterns and protein co-regulation.

Network analysis through String database revealed that AHSG and HPX are co-expressed with the other four proteins as shown in Fig. 6a,b. CRP does not co-express with AMBP and APOA4. The interaction networks between the proteins were based on text-mining and co-expression. Co-expression of these proteins was evident in humans as well as other organisms (rodents, fish, cows etc.), although variations were observed among the different species (Fig. 6c). Highest co-expression in *Homo sapiens* was between AHSG and AMBP, while the other proteins showed minimal co-expression levels.

**Figure 7:**
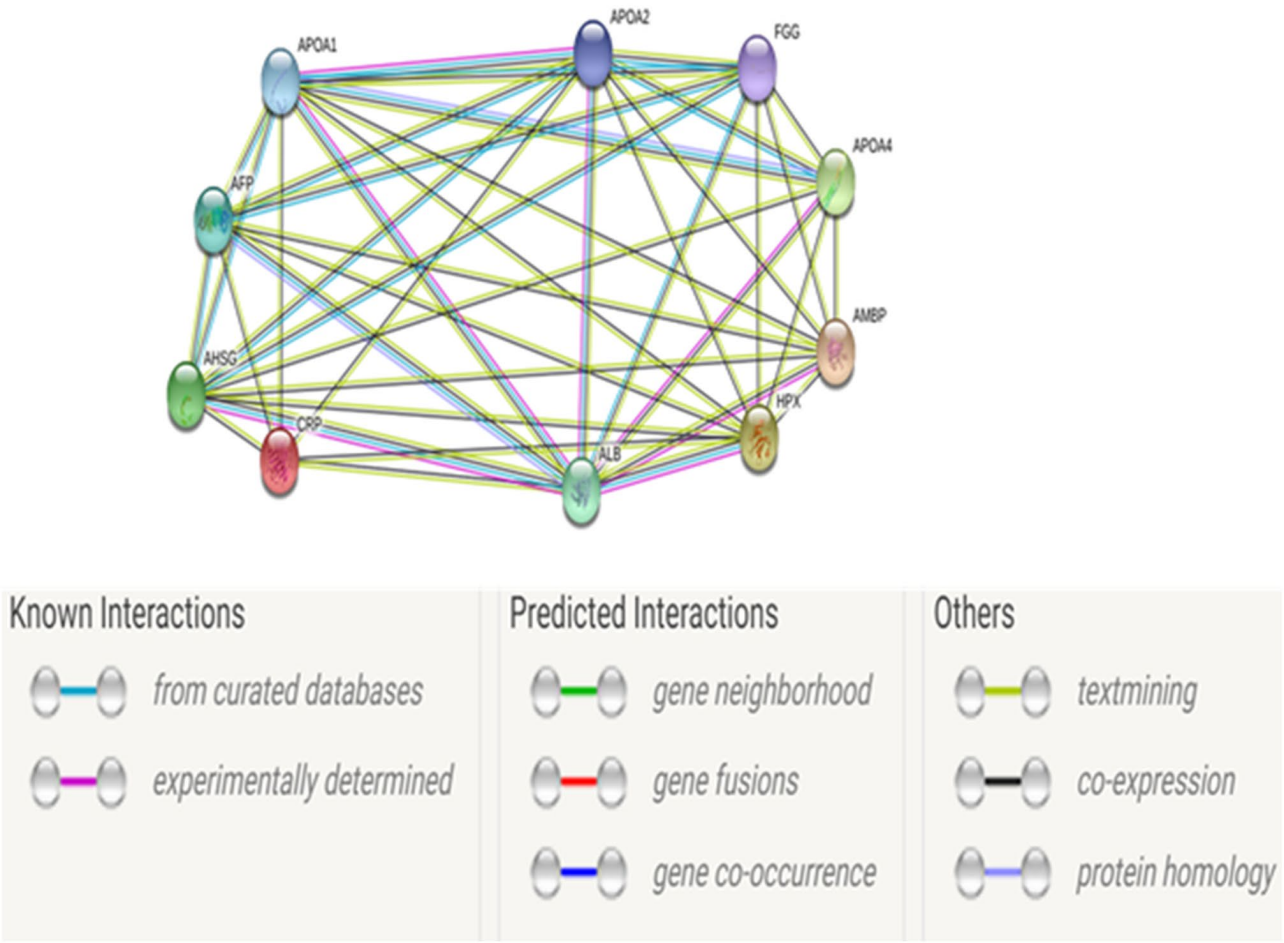
Network interactions between the selected proteins. The networks were generated in String database and show co-expression between the differentially expressed proteins and other unrelated proteins.

**Figure 8:**
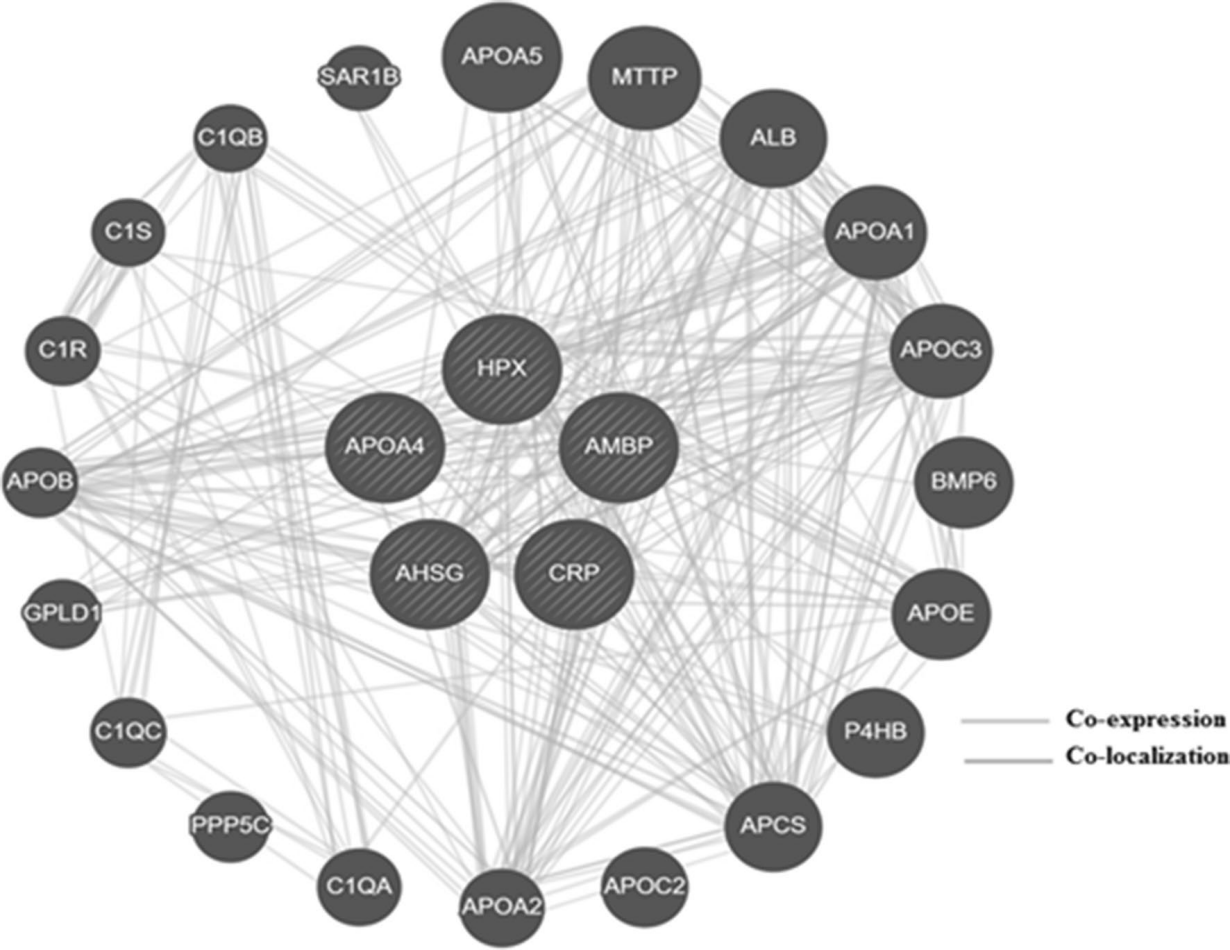
Genetic interactions among the selected proteins in humans. The co-expression and co-localization with other genes was observed through GeneMANIA.

Although no research has been done to study interactions among the selected proteins, through literature these proteins interact strongly with other proteins that are somehow associated with the differentially expressed proteins from this study. Albumin (ALB) has been proven to interact with all the proteins except CRP (Fig. 7). The expression of these proteins is strongly associated with alpha fetoprotein (AFP), APOA1, APOA2 and fibrinogen gamma chain (FGG). Of the five, only APOA1, APOA2 and ALB were also predicted by GeneMANIA software (Fig. 8); where their interactions are through co-localization or co-expression. The roles of these proteins in obesity and obesity related diseases are unclear, but they might be involved in a coordinated pathway leading to the development of obesity and its related conditions.

## Discussion

Predisposition of people to obesity is due to the interaction between genetic and environmental factors. Multiple studies have implicated high-calorie diets as the main environmental factor that triggers the development of obesity in genetically susceptible individuals^16,17^. Recently, there has been a rise in the number of studies that investigated the non-genetic, maternally-derived risk factor due to the rise in the prevalence of obesity in early life^18^. This information is owed to years of research on animal models of obesity, which strongly correlate to human obesity. Animal models of DIO mimic some features of human obesity and can be used to decipher obesity development and progression in a manner observed in human beings. Moreover, some of the key variables can be easily controlled and monitored with high reproducibility unlike in human studies^19,20^. The rat model used in this study has been reproducible over several experiments and displayed similar features with human obesity^21,22^. These rats gained body weight after eating the HF diets in just 8 weeks, had increased glucose levels (data not shown) and adipose tissue mass (Fig. 3) when compared with the rats fed the LF diet. This model proved to be appropriate to study factors responsible for the transition from lean to obese state and could be useful in attempts to decipher issues central to obesity development and progression. Literature have shown that 8 weeks of HF diet feeding was sufficient to induce obesity and significant differences in the blood proteome profile of rodents^23,24^.

Proteomics has become increasingly important in recent years, and this is driven by the hope of finding new molecular diagnostic biomarkers and potential therapeutic targets that are disease-specific in biological samples^25^. Blood is one of the easily accessible samples and a reliable source of disease indicators. Moreover, blood perfuses through different tissues and organs within the body, its protein profiles could be reflective of ongoing changes occurring within the body in response to physiological and pathological states^26^. Characterization of serum protein profiles could enable the discovery of reliable disease-specific biomarkers^24^, which can then be used in diagnosis, prognosis, and monitoring of disease progression. However, serum proteomics is complicated by the presence of high abundant proteins (HAPs) which constitute 95% of total protein content which obscure the detection of low abundant proteins (LAPs)^26,27^. The LAPs are made up of several classes of physiologically important proteins such as cytokines, chemokines, peptide hormones, and proteolytic fragments of larger proteins^26^. In order to avoid throwing away biologically important proteins, both the albumin-depleted and albumin-containing samples were studied. Several proteins were differentially expressed in the HF groups, of which five (CRP, AHSG, AMBP, HPX and ApoA4) were significantly dysregulated. At face value and studying the molecular functions of these proteins, it does not seem like there is any correlations between the proteins. Their biological functions are well documented. ApoA4 is regarded as a satiety signal and controls the food intake and body weight^28^. It is also involved in lipid transport, and therefore its expression is expected to increase as fat increases in the body^29,30^. In addition to these classical roles, ApoA4 has been shown to possess anti-atherogenic and anti-oxidant properties^31^. CRP is an acute-phase sensitive marker for systemic inflammation^32,33^, infection and tissue damage^34^. Elevated levels of CRP are common among individuals with both features of insulin resistance, overt T2D^35^, overweight, obesity^32^ and metabolic syndrome^33^. The physiological mechanisms linking elevated CRP to these disorders are still not clear, but their association is believed to be mediated by the adipose tissue^24^. Unfortunately, CRP is part of the nonspecific acute-phase response to many diseases and cannot be used on its own in disease diagnostics^34^. AHSG (Fetuin-A) is an abundant circulating blood protein that is produced mainly in the liver. It has diverse biological functions including regulation of calcium homeostasis and inhibition of insulin-induced autophosphorylation of the insulin receptor (IR) and IR-tyrosine kinase activity. The Fetuin-A gene has been identified as a susceptibility locus for T2D and metabolic syndrome^36^. HPX plays a role in recycling of iron within the bloodstream, it is an acute phase protein with anti-oxidant properties. The role of HPX in obesity is unclear, but it has been shown to be expressed by the adipose tissue and implicated in lipid metabolism. Its action in lipogenesis, adipogenesis and adiponectin expression could shed some insights on obesity development and progression^37^. Expression of AMBP was downregulated in diet-induced obese Sprague–Dawley rats^24^. However, its role in the development of obesity and progression to obesity-induced diseases is still vague. Therefore, there is a need to investigate whether the changes in its expression is due to the development of the disease instead of the effect of HF diet consumption.

Independent studies have implicated AMBP^24^, ApoA4, CRP, AHSG^38^ and HPX^23^ in DIO and diabetes. APOA4 was also increased in plasma samples of 10-year-old prepubertal overweight and obese boys^39^ an indication that there could be a relationship between obesity and altered expression of APOA4. AHSG and CRP expression were found to be increased in obese T2D model rats (OLETF rats) at 34 weeks of age when compared to their control (LETO rats)^38^. In this study, AMBP was significantly reduced at 8 weeks, and its expression at 42 weeks did not show any statistical significance. This may suggest that AMBP could be useful as an acute or an early biomarker for those at risk of developing obesity or other conditions associated with obesity. In a study by Zhao and colleagues, AMBP expression was also reduced at 8 weeks^24^. Differential expression of CRP and HPX, in addition to coagulation and complement factors, were also reported in human plasma samples obtained from overweight and obese individuals^40^. AHSG expression was upregulated in serum samples of stage IV obese breast cancer patients^41^. Although the blood proteome profiles reported for obese subjects does not entirely represent obesity-specific markers, they may be used as predictive biomarkers to identify individuals at risk of developing obesity comorbidities. These biomarkers can be used to detect the severity of obesity, from moderate to morbid state, and follow their progression to other chronic diseases, and thus serve as a preventative measure. Evidently, the available obesity-associated biomarkers, such as cytokines, are involved in other signalling processes and which makes it difficult to find molecules that are truly obesity-specific. For instance, leptin and TNF-α are pro-inflammatory cytokines and their increased expression is an indication of obese patients at higher risk of T2D and CVDs. Changes in expression of adiponectin and omentin are also associated with inflammation, CVDs and dysregulated glycemic control. CRP, sex hormone-binding globulin, complement factors and apolipoproteins are used as weight loss markers^40^. Obesity is a multifaceted disease and a risk factor for metabolic and CVDs^2–4^. As such, proteins identified in this study might be reflective of dysregulated processes, and not artefacts. Moreover, the use of diets that differ in their fat type and fatty acid composition revealed that the serum proteome can be modulated differentially by the diet, which supports the gene by environment interactions in the development of obesity. It is possible, as a limitation to this study, that other proteins that are differentially expressed in diet-induced obesity are not picked up due to the sensitivity of the technique^42,43^. The use of other techniques such as LC/MS/MS could potentially have provided other potential biomarkers that were expressed at low levels. The use of 2D PAGE has been reviewed by Abdallah et al.,^44^, whereby the limitations were mentioned to include issues related to reproducibility, poor representation of low abundant proteins, highly acidic/basic proteins, or proteins with extreme size or hydrophobicity, and difficulties in automation of the gel-based technique^44^. This is undoubtedly one of the shortfalls in this study. Nonetheless, the differentially expressed proteins from this study, together with their genes, could be useful targets that can help in the prevention of obesity and progression to chronic diseases that are associated with obesity. The feasibility of using these proteins as prognostic biomarkers was demonstrated in a clinical study of a diet-induced weight loss intervention for 8 weeks followed by weight maintenance for 12 months. Plasma protein profile changed and normalized after sustaining 12% of weight loss, APOA4, CRP and HPX were among the proteins that were studied^4^, which corroborated the findings of this study.

## Conclusion

In conclusion, this study showed differential expression of serum proteome in rats fed HF diets compared to rats fed LF diet. The reduced expression of AMBP in the HF fed groups during the early development of obesity requires further studies. Moreover, in-silico studies showed interactions between the identified proteins, and these will be pursued in future studies to elucidate the mechanisms and the roles played by these proteins. This will lead to potential therapeutic and preventive strategies to improve the management of obesity-associated chronic diseases.

## Methods

### Animal studies and diets

Male Wistar rats were obtained at weaning from the Primate Unit (South African Medical Research Council/ SAMRC, Tygerberg, South Africa). The rats were given standard rodent chow (low fat, LF) for two weeks during adaptation to the housing conditions. The animals were then bodyweight matched and divided into LF and HF groups. The rats were fed ad libitum on their specific diets with free access to fresh water for the duration of the study. The rats were singly caged in a temperature-controlled environment at 23 °C, and 12 h light/dark cycle (6:00 am/6:00 pm). The rats were weighed weekly after they started eating their respective diets. At termination, all animals were weighed and sacrificed. Various tissues were weighed, collected and stored at − 20 °C until further analysis. The use of animals was in line with the SAMRC and ARRIVE guidelines that recognize the NC3Rs initiative. All the procedures involving animals were approved by the South African Medical Research Council Ethics Committee for Research on Animals (SAMRC-ECRA) Ref: P04/10/021.

#### Induction of diet-induced obesity

Two independent studies were conducted. The first study was performed to determine the effects of three high-calorie diets on the development of obesity for 8 weeks, and the second study was to explore one diet that showed the highest rate of obesity induction for 42 weeks. The composition of the four diets was analyzed using Gas chromatography mass spectrometer (GC–MS).

##### Experiment 1

28 rats were bodyweight matched into four groups (n = 7 rats/group). One group served as the control (LF) group and was fed chow (Epol, South Africa), and the remaining three groups were fed three different chow-based HF diets to induce obesity for 8 weeks. Dietary composition of the four diets is shown in Table 3.

**Table 3:** dietary composition of LF and HF diets.

% Diet composition	Animal diets			
	LF	HF1	HF2	HF3
Fats	1.30	34.97	19.28	15.25
Protein	25.05	9.86	19.27	20.85
Carbohydrates	66.70	49.97	57.00	59.45
g/g diet	0.93	0.95	0.96	0.96
KJ/g diet	12.73	18.48	16.79	16.10
kcal/g diet	3.04	4.42	4.01	3.85

*LF* low fat, *HF* high fat.

##### Experiment 2

14 rats were body weight matched into LF and HF groups (n = 7). HF1 diet was used to induce obesity in the HF group, whereas the LF group remained on chow. The rats were fed their respective diets for 42 weeks.

#### Collection of blood samples

Blood samples were collected at 0, 8 weeks for the first study, as well as 42 weeks for the second study. On the day of blood collection for the baseline samples (0 weeks), food was removed for 4 h in the morning, in order to obtain postprandial state. Rats were lightly anaesthetized with 5% Halothane (Safe Life Pharmaceuticals, Sri Lanka), and blood samples (0.5 ml) were collected into Vacutainer serum tubes (Becton Dickinson, South Africa) by slightly snipping off the tip of the tail. At 8 and 42 weeks, blood samples were collected through a hepatic vein during exsanguination. The blood samples were left at room temperature for 30 min, followed by centrifugation at 7 000 rpm for 10 min at 4 °C. The supernatant (serum) was collected into new tubes and stored at − 80 °C until further analysis.

### Serum proteomics

#### Removal of high abundant proteins

The serum samples were precipitated to remove albumin following the TCA/acetone protocol described by Chen et al*.,*^7^ with some modifications. Four volumes of 10% TCA/acetone was added to the serum samples and incubated at − 20 °C for 90 min. The samples were centrifuged on an Eppendorf Refrigerated Microcentrifuge (Model 5417R, Sigma-Aldrich, MO, USA) at 14 000 rpm for 15 min at 4 °C. The supernatant was transferred into a new tube and kept on ice. The pellet was washed with 0.5 mL cold acetone and incubated for 15 min at − 20 °C, then centrifuged as before. The supernatants were combined, and 0.5 mL of fresh acetone was added and incubated at − 20 °C for 90 min and centrifuged as above. Both the TCA/acetone precipitated (albumin-depleted) and acetone precipitated (albumin-containing) pellets were air dried and resuspended in 0.2 mL of solubilization buffer (7 M urea, 2 M thiourea and 4% CHAPS; Sigma, USA). The protein concentrations were quantified by Bradford assay and the samples were stored at − 20 °C until further analysis.

#### Two-dimensional gel electrophoresis

Samples containing 100 µg protein were passively rehydrated onto 7 cm IPG strips (Bio-Rad, Hercules, CA, USA) with a pH range 4–7 for 12–16 h, followed by isoelectric focusing through the three-step protocol: the initial voltage was limited to 250 V for 10 min then increased to 3,500 V for 2,800 Vhr, then continued at 3,500 V to 3,700 Vhr. The current was limited to 50 µA per strip at 20 °C. After isoelectric focusing, the strips were first equilibrated in equilibration (EQ) buffer containing 2% 1,4-dithiothreitol (Bio-Rad, Hercules, CA, USA) for 15 min, followed by another 15 min equilibration in the EQ buffer containing 2.5% Iodoacetamide (Bio-Rad, Hercules, CA, USA) with shaking. The proteins were resolved on a 10% SDS-PAGE for the albumin-containing samples and 12% SDS-PAGE for the albumin-depleted samples. The gels were fixed in methanol/acetic acid/water solution (4:1:5) for 2 h, followed by 3 h of staining with 1X Flamingo fluorescent stain (Bio-Rad, Hercules, CA, USA).

#### Quantitative analysis of gel images

The gel images from both the LF and HF groups were scanned on a Molecular Imager Pharos FX system (Bio-Rad, Hercules, CA, USA). Image analysis including image editing, spot finding, quantitation, and matching, was carried out using Quantity One Software (Bio-Rad) and PD Quest version 8.0 2-D gel analysis software (Bio-Rad, Hercules, CA, USA). The protein spots were detected by following the PD Quest software instructions using the following parameters: horizontal and vertical streaking removal with a radius of 33, smoothing by Power Mean filter with kernel size 3 × 3, speckle removal at a sensitivity of 50. The densities of protein spots were normalized using the Local Regression Model. The spots were then quantitatively compared between LF and HF groups using the approach by Sun et al.^43^. Protein spots were considered to be differentially expressed if the difference between the average of spot densities between the two groups was twofold greater or lesser.

#### In gel proteolysis

The protein spots that were differentially expressed between the LF and HF groups were excised from the gels using the EX Quest spot cutter (Bio-Rad, Hercules, CA, USA). The protein spots were tryptically digested following the protocol adapted from Shevchenko^42^. The Proteomics Analyzer, Voyager DE PRO MALDI MS (Applied Biosystems, UK) was used to identify the obtained mass spectra that were searched through a Mascot search engine (https://www.matrixscience.com) then queried against the National Centre for Biotechnology Information (NCBI) protein database for protein identification.

#### Western blot analysis

The identities of the differentially expressed proteins were validated by western blotting. The protein samples, 20 µg for albumin-depleted sample and 10 µg for albumin-containing sample, were resolved on a 12% SDS-PAGE. The proteins were electroblotted onto Hybond-P nitrocellulose membrane (Amersham Biosciences, UK). The membranes were incubated overnight at 4 °C with 1:5,000 primary antibodies (Apo A-IV, CRP, Fetuin-A and transferrin (TFR); Santa Cruz Biotechnology, TX, USA), washed and incubated with 1:10 000 secondary antibodies conjugated to horseradish peroxidase for 1 h at room temperature. The membranes were developed with LumiGLO Chemiluminescent Substrate System (Whitehead Scientific) then exposed to X-ray film (Amersham Biosciences) for 5 min in a dark room. The X-ray films were processed using the X-ray processor (Agfa, South Africa), and the images were captured using a Digital Camera. Transferrin was used as a loading control. The X-ray films were scanned with a Molecular Imager Pharos FX scan. Image analysis including image editing and quantitation was carried out using ImageJ Software (National Institute of Health).

#### Network analysis

Interaction between selected proteins were analysed through String v10.5 (https://string-db.org) and GeneMANIA (https://genemania.org). The following gene codes: APOA4, AMBP, AHSG, CRP and HPX were used to determine how these proteins interact with each other at gene and protein levels.

### Statistical analysis

The animal body weights were analyzed statistically using one-way analysis of variance (ANOVA) followed by Dunnett’s post hoc analysis performed using GraphPad Prism version 5.00 for Windows, GraphPad Software, La Jolla Carlifonia USA, www.graphpad.com. The results were presented as means ± SEM. Statistical analysis for 2D gel analysis, the comparison between LF and HF groups was assessed using Student’s *t*-test and Boolean analysis sets by the PD Quest software (Bio-Rad, South Africa). The differences were considered statistically significant at *p* < 0.05.

## Supplementary information

Supplementary Information.

## Abbreviations

AFP: Alpha fetoprotein
AHSG: Alpha 2-HS glycoprotein
ALB: Albumin
AMBP: Alpha 1 macroglobulin
APOA4: Apolipoprotein-AIV
CRP: C-reactive protein
CVDs: Cardiovascular diseases
DIO: Diet-induced obesity
FAs: Fatty acids
FGG: Fibrinogen gamma chain
GC–MS: Gas chromatography mass spectrometer
HF: High fat
HPX: Hemopexin
LF: Low fat
MALDI-MS: Matrix assistant laser desorption/ionization mass spectrometry
SDS-PAGE: Sodium dodecyl sulfate polyacrylamide gel electrophoresis
T2D: Type 2 diabetes
TCA: Trichloroacetic acid
