# Supplementary material for: Differentially expressed serum proteins from obese Wistar rats as a risk factor for obesity-induced diseases
Source: Sci Rep. 2020 Jul 24;10:12415. doi: 10.1038/s41598-020-69198-2 (PMC7381623; doi:10.1038/s41598-020-69198-2)
Supplement: Supplementary file 1 — Supplementary Information. [file 41598_2020_69198_MOESM1_ESM.pdf]

SUPPLEMENTARY DATA

Gabuza et al

Differentially Expressed Serum Proteins from Obese Wistar Rats as a risk factor for obesity-induced diseases

Kwazikwakhe Bethuel Gabuza[1]

Nicole Remaliah Samantha Sibuyi[1]

Mmabatho Peggy Mobo[1]

Abram Madimabe Madiehe[1,*]

[1]Department of Biotechnology, University of the Western Cape, Bellville, Cape Town, South Africa

Correspondence: Abram Madimabe Madiehe

Department of Biotechnology

University of the Western Cape

Bellville, 7535

South Africa

Tel +27 21 9592468

Fax +27 21 9593505

Email: amadiehe@uwc.ac.za

Image analysis of the 2D PAGE was performed using the parameters detailed in the methods section of the manuscript. Quantity One software was used for image capturing and the images that were captured were analysed using PD Quest. The data output is presented in scatter plots Fig S1.

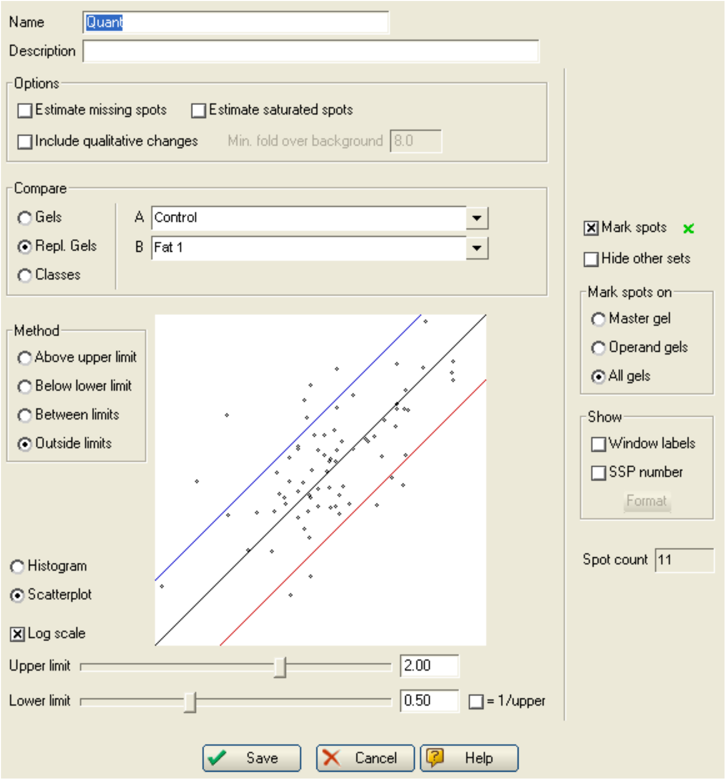

Figure S1: The scatter plot that shows the differential expression from 0.5 fold (black middle line) to two fold up-regulated (blue line) and two fold down-regulated (red line), as obtained from the PD Quest analysis of different gels between the control (LF) and groups on high fat diet (HF).

The verification of the expression obtained from 2D gel analysis was verified by Western blot performed in the albumin depleted samples on an SDS-PAGE. The full blots are presented as AHSG, APOA4, CRP and TRF (Figure S2 a-c).

a. b. c.

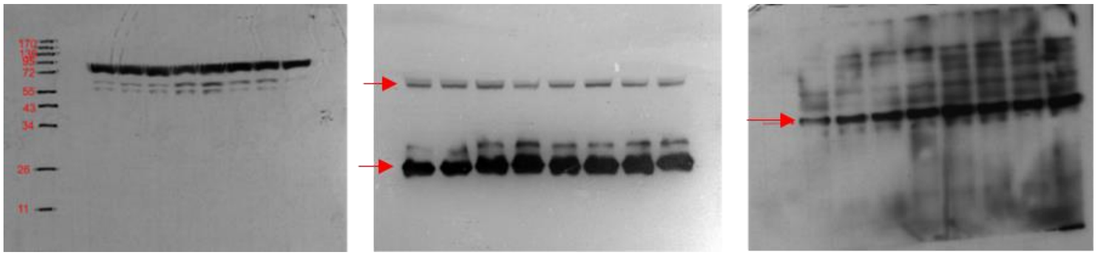

Figure S2: Full blots for AHSG (a), CRP (bottom band with an arrow pointed at it) and TFR (top band with an arrow pointing at it (b). The third blot is for APOA4 (c). The blots were imaged from the films using the ChemiDoc MP (Bio-Rad, Hercules, CA, USA).
